# Hidden symmetry and protection of Dirac points on the honeycomb lattice

**DOI:** 10.1038/srep17571

**Published:** 2015-12-07

**Authors:** Jing-Min Hou, Wei Chen

**Affiliations:** 1 Department of Physics, Southeast University, Nanjing 211189, China; 2 College of Science, Nanjing University of Aeronautics and Astronautics, Nanjing 210016, China

## Abstract

The honeycomb lattice possesses a novel energy band structure, which is characterized by two distinct Dirac points in the Brillouin zone, dominating most of the physical properties of the honeycomb structure materials. However, up till now, the origin of the Dirac points is unclear yet. Here, we discover a hidden symmetry on the honeycomb lattice and prove that the existence of Dirac points is exactly protected by such hidden symmetry. Furthermore, the moving and merging of the Dirac points and a quantum phase transition, which have been theoretically predicted and experimentally observed on the honeycomb lattice, can also be perfectly explained by the parameter dependent evolution of the hidden symmetry.

Graphene, a honeycomb lattice of carbon atoms, has attracted extraordinary attention in the last decade, due to its remarkable properties and potential applications^1^,^2^,^3^,^4^. The band structure of this exotic material is characterized by two distinct Dirac points in the Brillouin zone, dominating most of its physical results. Although there are a multitude of researches on graphene and other honeycomb lattices^5^,^6^,^7^,^8^,^9^,^10^,^11^,^12^,^13^, the origin of the Dirac points is still unclear. According to the von Neumann-Wigner theorem^14^,^15^, there must be some symmetry to protect the Dirac points on the honeycomb lattice, while the robustness of Dirac points during the deformation of the lattice structure^16^ excludes the possibility of a point group protection. The time-reversal and inversion symmetries are known to stabilize the Dirac points on the honeycomb lattice in a limited parameter range, i.e., once they are there, the Dirac points cannot be destroyed by small perturbations preserving those symmetries^17^,^18^. However, these symmetries are not sufficient to guarantee the existence of the Dirac points on the honeycomb lattice (see supplementary information), because strong perturbations preserving these symmetries can induce the annihilation of Dirac points^19^,^20^,^21^,^22^,^23^. As a result, a novel symmetry is expected to be responsible for the Dirac points.

In this paper, we unveil the mysterious story behind the Dirac points by showing that they are exactly protected by a kind of hidden symmetry on the lattice structure. As its name suggests, the hidden symmetry is not so obvious as usual symmetries such as the point group symmetry. In general, it can be described by a composite antiunitary operator, which consists of a translation, a complex conjugation, and a sublattice exchange, and sometimes also a local gauge transformation and a rotation, or is the extension of the composite operator by a mapping method. This kind of symmetry is seldom studied before and was firstly discovered by one of the authors in a toy model^24^,^25^. We find that the hidden symmetry on the honeycomb lattice evolves along with the variation of the parameters, which can perfectly explain the moving and merging of the Dirac points and the quantum phase transition on the honeycomb lattice that have been theoretically predicted^19^,^20^,^21^,^22^,^23^ and experimentally observed^16^.

## Results

### Model

**Figure 1:**
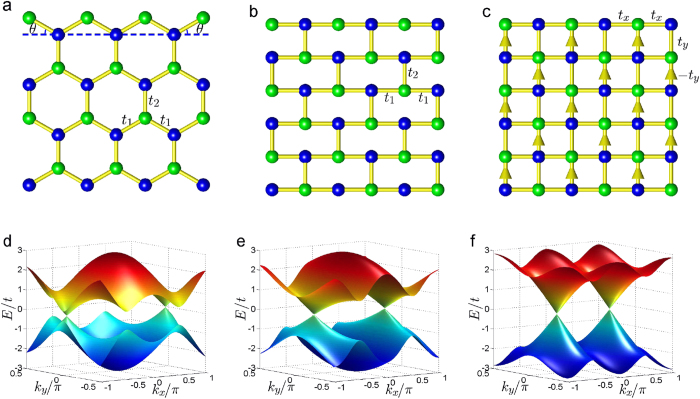
The lattices and the dispersion relations. (**a**) Schematic of the honeycomb lattice. *θ* denotes the angle between the bonds on the zigzag line and the horizontal direction; 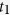 and 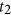 represent the amplitudes of hopping. (**b**) Schematic of the brick-wall lattice, which can be regarded as a special case of the honeycomb lattice with *θ* = 0. (**c**) Schematic of the square lattice. The arrows represent a hopping-accompanying *π* phase; 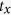 and 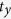 represent the amplitudes of hopping. In (**a**), (**b**,**c**), the blue and green balls represent the lattice sites in sublattices A and B, respectively. (**d**) The dispersion relation for the honeycomb lattice model with 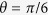 and 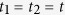. (**e**) The dispersion relation for the brick-wall lattice model with 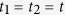. (**f**) The dispersion relation for the square lattice model with 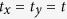.

To be specific, we consider the general honeycomb lattice as shown in Fig. 1(a), where we define a bond angle *θ* as the angle between the bonds on the zigzag line and the horizontal direction. The general honeycomb lattice model with the bond angle *θ* can be well described by the Bloch Hamiltonian as (the unit bond length is adopted)


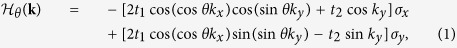


where 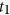 and 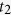 denote the amplitudes of hopping as sketched in Fig. 1(a); 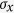 and 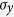 are the pauli matrices defined in the sublattice space (for detail see supplementary information). Diagonalizing equation (1), we obtain the dispersion relation as


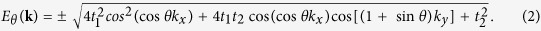


Concretely, when 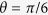, the lattice is the ideal honeycomb lattice, such as graphene, which has the Bloch Hamiltonian as


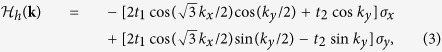


and the dispersion relation as 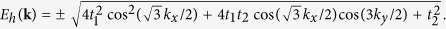 When *θ* = 0, the lattice reduces to the brick-wall lattice as shown in Fig. 1(b), which has the corresponding Bloch Hamiltonian as


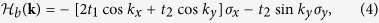


and the corresponding dispersion relations as 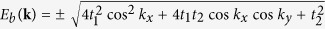. We find that, for the ideal honeycomb lattice and the brick-wall lattice with the parameters 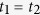, the band structures are both gapless and have the Dirac points at 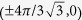 and 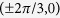 in the Brillouin zones (for the definitions of the Brillouin zones see Methods) as show in Fig. 1(d,e), respectively.

In order to find the hidden symmetry behind the the honeycomb lattice, an auxiliary square lattice with a hopping-accompanying *π* phase is introduced as well, as shown in Fig. 1(c), which has an intrinsic relation with the honeycomb lattice. The Bloch Hamiltonian for the square lattice can be written as


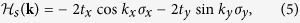


where 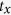 and 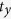 represent the amplitudes of hopping along the horizontal and vertical directions, respectively (for detail see supplementary information). The corresponding dispersion relation is


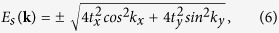


which is gapless and has Dirac points at 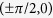 in the Brillouin zone as shown in Fig. 1(f).

Geometrically, the square lattice can transform into the ideal honeycomb lattice continuously in two steps. First, the square lattice changes into the brick-wall lattice when the amplitude of hopping with a *π* phase is tuned to zero, and then reaches the ideal honeycomb lattice by a deformation of the bond angle *θ* from 0 to 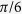, which can be understood with the help of Fig. 1(a–c). Besides the intuitive relation between these lattice structures in the real space, their band structures also strongly correlate to each other. The energy bands are all characterized by two linear Dirac cones in the Brillouin zone as shown in Fig. 1(d–f). More importantly, these Dirac points are able to evolve continuously into each other with the variation of the lattice parameters. For the general honeycomb lattice with *β* = 1 (*β* is defined as the hopping amplitude ratio 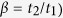, the corresponding Dirac points locate at 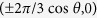 in the Brillouin zone. As a result, when the bond angle *θ* varies from 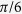 to 0, the lattice first changes from the ideal honeycomb lattice into the brick-wall lattice, inducing a shift of the Dirac points from 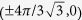 to 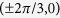, as shown in Fig. 1(d,e). Starting from the brick-wall lattice, the square lattice can be obtained by turning on the amplitude of hopping with a *π* phase from 0 to 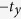. Accordingly, the corresponding Dirac points evolve from 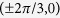 to 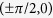, as shown in Fig. 1(f). It is impressive that during the whole evolution of the lattice, the Dirac points are always stable without any gap opening. We will show that this property can be perfectly explained by the protection of the hidden symmetry of the lattice structures.

### Hidden symmetry on the auxiliary square lattice and protection of Dirac points

**Figure 2:**
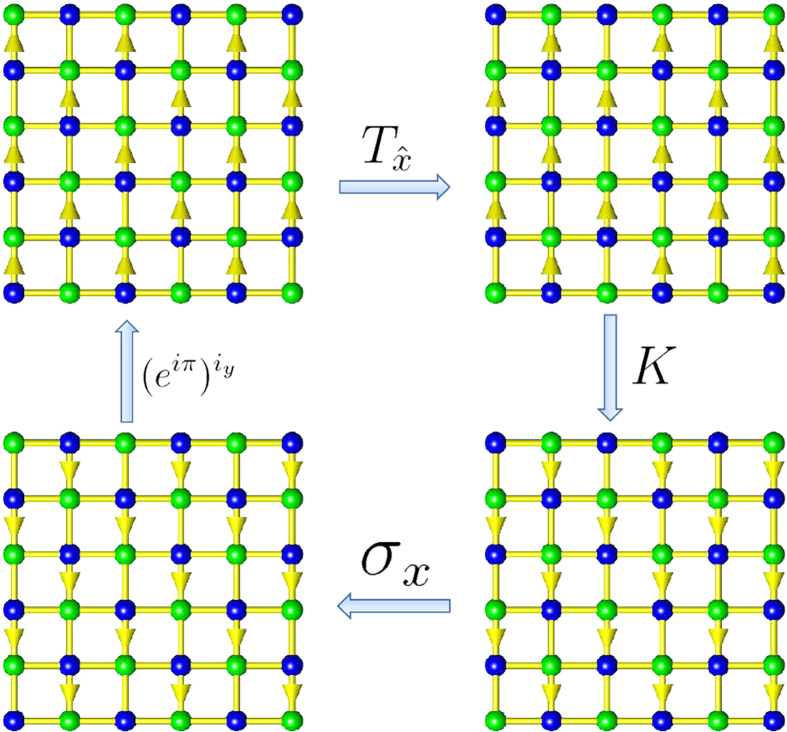
Schematic of the invariance of the square lattice under the action of the hidden symmetry ϒ. The hidden symmetry consists of a translation transformation 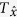, a complex conjugation *K*, a sublattice exchange 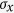 and a local 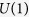 gauge transformation 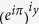 in order. Here, the arrows represent a hopping-accompanying *π* phase.

Firstly, we consider the auxiliary square lattice as shown in Fig. 1(c). One can verify that the square lattice is invariant under the action of the operator defined as


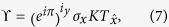


where 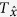 is a translation operator that moves the lattice along the horizontal direction by a unit vector 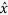; *K* is the complex conjugate operator; *σ*_*x*_ is the Pauli matrix representing the sublattice exchange; 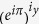 is a local 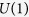 gauge transformation and 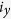 is the *y* component of the coordinate of lattice sites. Figure 2 schematically shows the invariance of the square lattice under the action of the hidden symmetry ϒ. This kind of transformation invariance indicates a hidden symmetry of the square lattice^24^. It is easy to prove that the symmetry operator ϒ is antiunitary, and its square is equal to 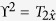.

Mathematically, the hidden symmetry operator ϒ can be considered as a self-mapping of the square lattice model defined as


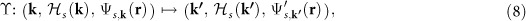


where 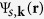 and 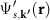 are the Bloch functions of the square lattice model. We suppose that the Bloch functions of the square lattice model have the form as


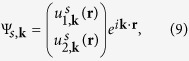


where 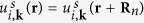 with 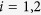. Then, the hidden symmetry operator ϒ acts on the Bloch functions as follows


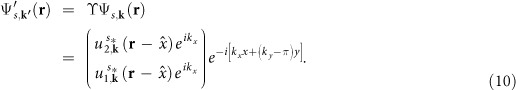


Comparing equation(10) with equation(9), we have 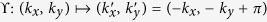, which can be regarded as the transformation of the wave vector under the action of the operator ϒ. If 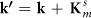, where 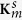 is a reciprocal lattice vector for the square lattice, then we can say that 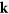 is a 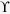-invariant point. In the Brillouin zone, the ϒ-invariant points are 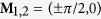 and 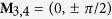. After the hidden symmetry operator acts on the Bloch function twice, we have 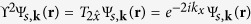. We define 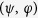 as the inner product of the two wave functions 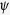 and 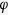. The antiunitary operator 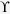 has the property that 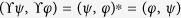. Therefore, at the 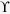-invariant point 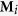 in the Brillouin zone, we have


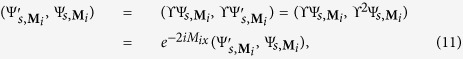


where 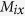 is the *x* component of the ϒ-invariant point 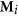 and the input of the Bloch functions is omitted for convenience. For the ϒ-invariant points 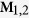, where 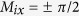, we have 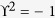, then we obtain the solution 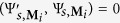, that is to say, 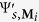 and 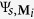 are orthogonal to each other. While, for the ϒ-invariant points **M**_3,4_, where 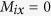, we have 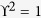, so 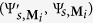 is unconstrained for equation (11). Therefore, we arrive at the conclusion that the system must be degenerate at points 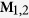, which are just the locations of the Dirac points as shown in Fig. 1(f). From the above discussion, one can see that the Dirac points on the auxiliary square lattice are exactly protected by the hidden symmetry ϒ.

### Mapping from the honeycomb lattice into the square lattice

In this section, we define a mapping from the honeycomb lattice into the auxiliary square lattice. In order to interpret this mapping in an intuitive way, we divide it into two mappings. The first one is the mapping from the general honeycomb lattice model with the bond angle *θ* into the brick-wall lattice model and the second one is the mapping from the brick-wall lattice model with the hopping amplitude ratio *β* into the square lattice model. In the following, we explain these in detail.

#### The mapping *ω*_1,*θ*_ from the honeycomb lattice into the brick-wall lattice

The general honeycomb lattice model with the bond angle *θ* is equivalent with the brick-wall lattice model in some sense. To manifest this equivalence, we define a mapping 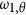 from the general honeycomb lattice model into the brick-wall lattice model as


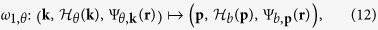


where 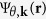 and 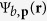 are the Bloch functions of the honeycomb lattice with the bond angle *θ* and the brick-wall lattice, respectively. To find the explicit form of the mapping, we take a transformation on the Bloch Hamiltonian (equation(1)) as 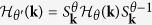, where the transformation matrix 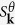 is defined as


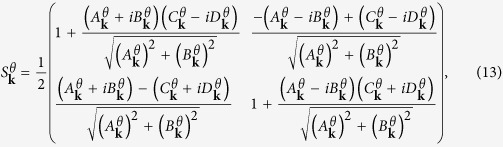


with 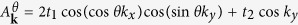, 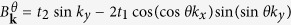, 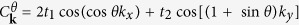 and 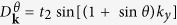, satisfying the identity 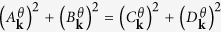. When 
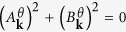
, the transformation matrix 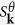 is ill-defined. For the continuity of the mapping, when 
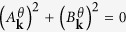
, we take the limit as the definition of 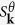. After the transformation, we then obtain


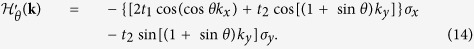


Substituting 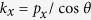 and 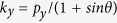 into 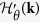, we obtain


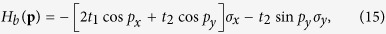


which is just the Bloch Hamiltonian (equation(4)) of the brick-wall lattice model. The mapping 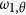 has the effect on the Bloch functions and the wave vectors as 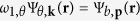 and 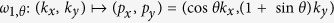. This mapping is one-to-one and surjective. Thus, we can regard this mapping as a kind of equivalence. The explicit form of the mapping depends on the bond angle *θ*. When *θ* = 0, this mapping is an identity mapping.

**Figure 3:**
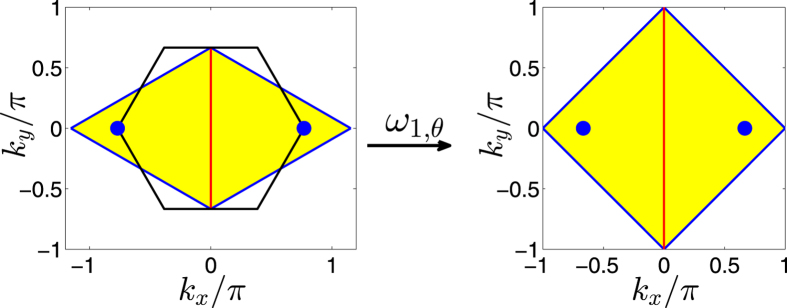
The mapping from the Brillouin zone of the honeycomb lattice into the Brillouin zone of the brick-wall lattice. The yellow areas in the left and right panels represent the Brillouin zones for the honeycomb lattice with 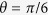 and the brick-wall lattices, respectively. Concretely, the blue lines, the red line, and the blue filled circles (the 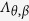-invariant points) in the left panel map into the blue lines, the red line, and the blue filled circles (the 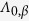-invariant points) in the right panel, respectively.

The mapping 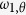 gives a one-to-one correspondence between the Brillouin zones of the honeycomb lattice with the bond angle *θ* and the brick-wall lattice. That is to say, for some wave vector **k** in the Brillouin zone of the honeycomb lattice, the Bloch Hamiltonian 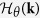 and its Bloch functions 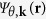, there correspondingly exist a wave vector **p** in the Brillouin zone of the brick-wall lattice, Bloch Hamiltonian 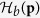, and Block functions 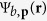. The mapping from the Brillouin zone of the honeycomb lattice with 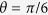 into that of the brick-wall lattice is schematically shown in Fig. 3.

#### The mapping *ω*_2,*β*_ from the brick-wall lattice into the square lattice

Similarly, we can define a mapping from the brick-wall lattice model to the square lattice model as


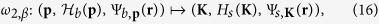


where 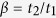 is the hopping amplitude ratio of the brick-wall lattice; 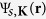 is the Bloch functions of the square lattice model. For this mapping, we have 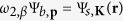 with


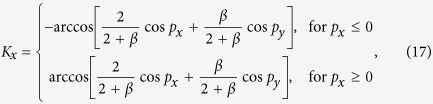



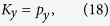


where **p** is a wave vector in the Brillouin zone of the brick-wall lattice. Through the mapping 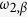, the Bloch Hamiltonian of the brick-wall lattice (15) becomes the form as follows


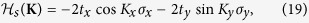


with 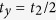 and 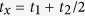, which is just the Bloch Hamiltonian of the square lattice model. The explicit form of this mapping depends on the hopping amplitude ratio *β*.

**Figure 4:**
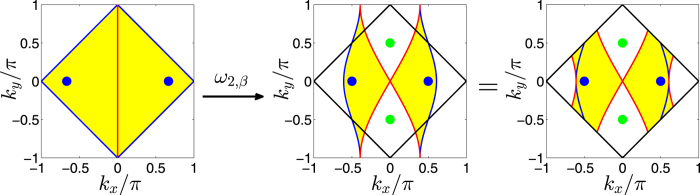
The mapping from the Brillouin zone of the brick-wall lattice into the Brillouin zone of the square lattice. The left panel shows the Brillouin zone of the brick-wall lattices; the middle panel shows the image of the mapping 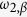 for the Brillouin zone of the brick-wall lattice in the reciprocal space of the square lattice; the right panel shows the image of the mapping 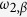 for the Brillouin zone of the brick-wall lattice restricted in the Brillouin zone of the square lattice. Here, the left and right half of the Brillouin zone of the brick-wall lattice in the left panel map into the the left and right yellow shadow areas in the middle panel, which is equivalent with that in the right panel. The blue and red lines in the left panel for the brick-wall lattice map into the blue and red lines in the middle panel for the square lattice, respectively. The blue filled circles (the 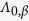-invariant points) in the left panel map into the degenerate 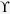-invariant points (blue filled circles in the middle and right panels), where 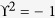, in the Brillouin zone of the square lattice. The green filled circles represent the 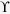-invariant points where 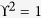 and no symmetry-protected degeneracy occurs for the square lattice.

This mapping is not surjective. That is to say, the image of the mapping for the Brillouin zone of the brick-wall lattice just covers part of the Brillouin zone of the square lattice. The mapping for the wave vectors is schematically shown in Fig. 4. In Fig. 4, the left panel shows the Brillouin zone of the brick-wall lattice. In order to clearly manifest the mapping from the brick-wall lattice model to the square lattice model, we first map the Brillouin zone of the brick-wall lattice into the reciprocal space of the square lattice, not restricted in the Brillouin zone, as shown in the middle panel of Fig. 4. The image of the Brillouin zone of the brick-wall lattice in the reciprocal space of the square lattice looks like a butterfly. The left and right halves of the Brillouin zone of the brick-wall lattice map into the left and right wings of the butterfly, respectively. If we restrict the image of the mapping in the Brillouin zone of the square lattice, then the butterfly-like image is equivalent to that as shown in the right panel of Fig. 4.

#### The composite mapping Ω_*θ*,*β*_

**Figure 5:**
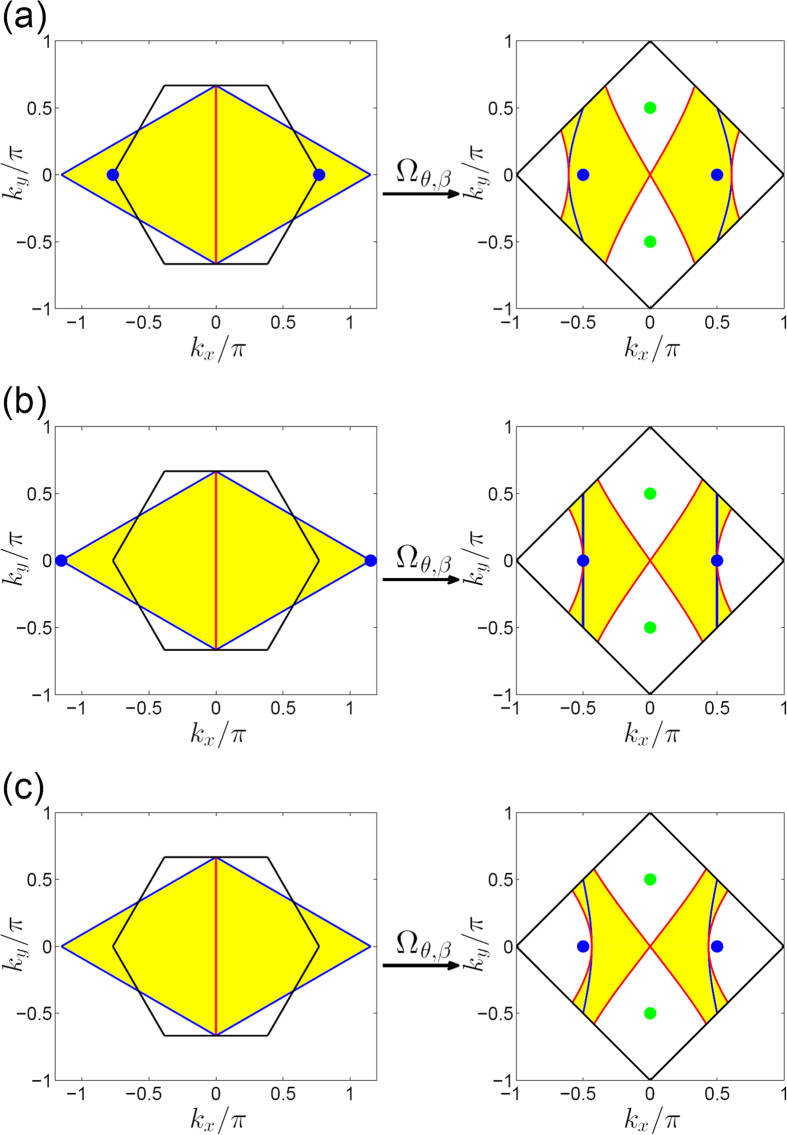
The mapping from the Brillouin zone of the honeycomb lattice into the Brillouin zone of the square lattice. (**a**) The case with 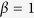. (**b**) The case with 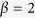. (**c**) The case with 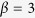. In the left panels, the yellow diamond areas represent the Brillouin zone of the honeycomb lattice, which are equivalent to the areas enclosed the black solid lines; the blue filled circles represent the 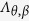-invariant points 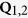. In the right panels, the square areas enclosed by the black solid lines represent the Brillouin zone of the square lattice; the blue and green filled circles represent the 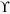-invariant points 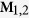 and 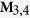, respectively; the yellow areas are the image of the mapping 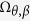 for the Brillouin zone of the honeycomb lattice. The mapping 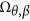 concretely map the blue filled circles, the blue and red lines in left panels into the blue filled circles, the blue and red lines in the right panels, respectively.

The above two mapping can be combined into a composite mapping 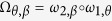, which is the mapping from the honeycomb lattice model into the square lattice model as


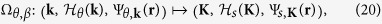


which depends on the bond angle *θ* and the hopping amplitude ratio *β*. For this mapping, we have 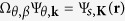 with


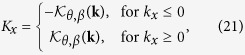



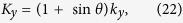


where 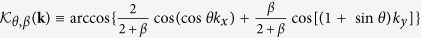. For the Bloch Hamiltonian, this mapping can be performed in the following manner. First, one takes a transformation on the Bloch Hamiltonian (1) as 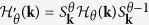, where the transformation matrix 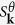. Second, replacing the wave vectors **k** in 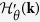 with **K** via equations(21) and (22), one obtains the Bloch Hamiltonian as


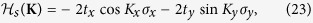


with 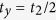 and 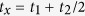, which is just the Bloch Hamiltonian of the square lattice model (i.e., equation(5)). This composite mapping is also not surjective, which can be shown in Fig. 5.

### Hidden symmetry on the honeycomb lattice and protection of Dirac points

With the help of the mapping 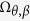, we define a symmetry operator of the honeycomb lattice as 
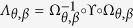
, which is a composite operation consisting of firstly mapping the honeycomb lattice into the square lattice, then the ϒ-transformation on the square lattice, and finally inverse mapping back to the honeycomb lattice from the square lattice. Therefore, the operation 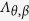 can be considered as a self-mapping of the honeycomb lattice model with the bond angle *θ* as


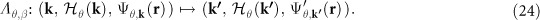


Applying this operator to the wave function, we obtain


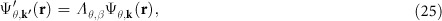


which is also the Bloch function of the honeycomb lattice model. After the action of the operator 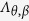, the wave vector **k** becomes


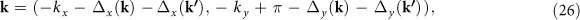


where


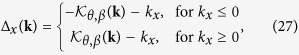



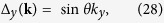


represent the shift of the wave vector **k** due to the mapping 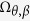. If


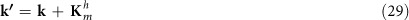


is satisfied, where 
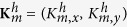
 is a reciprocal lattice vector of the honeycomb lattice, **k** is a 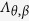-invariant point in the Brillouin zone of the honeycomb lattice with the bond angle *θ*. We assume 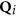 is a 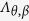-invariant point in the Brillouin zone. Substituting equation (26) and 
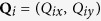
 into equation (29), we obtain the following equation


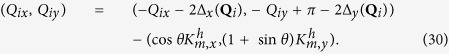


Solving the above equation, we obtain the 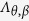-invariant points in the Brillouin zone are 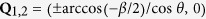.

It is straightforward to verify that 
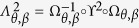
. Therefore, we have 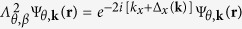. At the 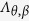-invariant point 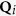, we have the following equation:


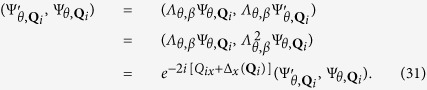


Substituting 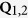, we obtain 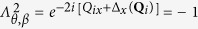 at 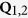. Thus, we have the solution 
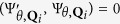
, which implies that 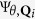 and 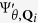 are orthogonal to each other. We can conclude that there must be the band degeneracy at the points 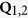. In particular, when 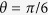 and *β* = 1, the 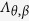-protected degenerate points are 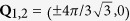, which are just the positions of the Dirac points on the ideal honeycomb lattice, such as graphene. When *θ* = 0 and *β* = 1, the 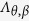-protected band degeneracies occur at 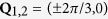, which correspond to the locations of the Dirac points on the brick-wall lattice.

### The explanation of moving and merging of Dirac points on the honeycomb lattice

We have proved above that the Dirac points on the honeycomb lattice are protected by the hidden symmetry 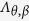. More generally, the moving and merging of Dirac points on the honeycomb lattice, which has been predicted theoretically^19^,^20^,^21^,^22^,^23^ and observed experimentally^16^, can also be explained by the hidden symmetry. Since the hidden symmetry operator 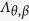 contains the parameters *θ* and *β*, the locations of the 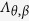-protected Dirac points, 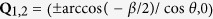, are also functions of the two parameters. As the hopping amplitude ratio 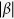 starts to vary, the Dirac points start to move. When 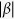 reaches 2, two Dirac points merge into one isolated degenerate point at the corner or the center of the Brillouin zone. If 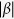 increases further, there is no solution to the 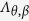-invariant points, thereby the Dirac points vanish, with a gap opening simultaneously. As a result, 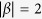 is the critical point of the quantum phase transition.

We can interpret the above conclusion in a more intuitive way by mapping the Brillouin zone of the honeycomb lattice into that of the square lattice, as shown in Fig. 5. It turns out that such a mapping is not surjective, which means that the image of the Brillouin zone of the honeycomb lattice is part of the Brillouin zone of the square lattice. In the parameter interval of 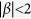, the image covers the 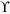-protected degenerate points 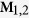 in the Brillouin zone of the square lattice, as shown in Fig. 5(a). Thus, there always exist two points 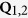 in the Brillouin zone of the honeycomb lattice mapping into the 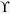-protected degenerate points 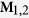 in the Brillouin zone of the square lattice. When 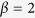, the two equivalent points locating at the corners at the center of the Brillouin zone of the honeycomb lattice map into the 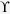-protected degenerate points 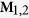, as shown in Fig. 5(b). Similarly, when 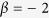, the isolated point locating at the center of the Brillouin zone of the honeycomb lattice map into the 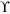-protected degenerate points 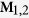. Therefore, the Dirac points on the honeycomb lattice merge. When 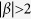, the image of the Brillouin zone of the honeycomb lattice can not cover the 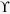-protected degenerate points 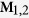, as shown in Fig. 5(c). Therefore, there is no point in the Brillouin zone of the honeycomb lattice mapping into the 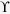-protected degenerate points 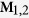. Thus, the Dirac points disappear and a gap opens.

We note that, due to the non-surjection of the mapping 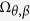, for some special case in the transformation 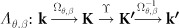, 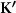 is out of the image of the mapping 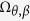, the inverse mapping 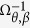 of 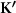 does not exist, which implies that **K** is not a 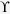-invariant points in the Brillouin zone of the square lattice, and corresponding **k** is not a 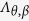-invariant points in the Brillouin zone of the honeycomb lattice. Thus, this singularity does not cause any problem for finding the 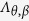-invariant points in the Brillouin zone of the honeycomb lattice. Inversely, the non-surjection of the mapping 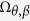 is necessary to explain the merging of the Dirac points on the honeycomb lattice.

## Discussion

In summary, we have found a hidden symmetry on the honeycomb lattice and proved that the hidden symmetry protects the Dirac points on the honeycomb lattice. The hidden symmetry evolves along with the parameters, such as the bond angle *θ* and the hopping amplitude ratio *β*, which provides a perfect explanation on the moving and merging of the Dirac points and the quantum phase transition on the honeycomb lattice. Our research unfolds a new perspective on the symmetry protected band degeneracy, which is totally different from the conventional ones, such as the band degeneracy protected by point groups or time reversal symmetry. Such novel hidden symmetry can greatly enrich and deepen our understanding of the band degeneracy, which will have important applications in modern condensed matter physics, especially, in the topics of Dirac (Weyl) semimetals and other topological semimetals.

## Methods

### The definitions of Brillouin zones

**Figure 6:**
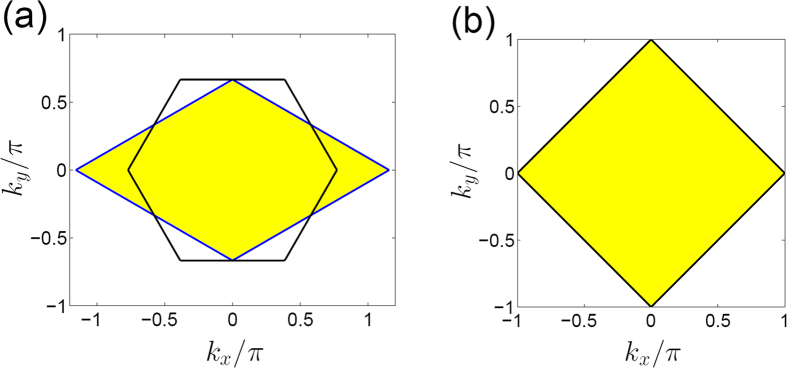
The Brillouin zones. (**a**) The Brillouin zone of the honeycomb lattice. The Brillouin zone of the honeycomb lattice can be represented by the hexagon area enclosed by the black solid lines, equivalently, it can also be represented by the yellow diamond. (**b**) The Brillouin zone of the brick-wall lattice and the square lattice.

For the general honeycomb lattice with the bond angle *θ*, the primitive lattice vectors are 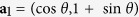 and 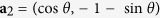. The primitive reciprocal lattice vectors are 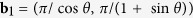 and 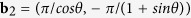. For 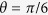 case, such as graphene, the symmetric Brillouin zone is hexagon, i.e., the area enclosed by the black lines in Fig. 6(a). An alternative Brillouin zone equivalent to the symmetric Brillouin zone is a diamond, i.e., the yellow shaded area in Fig. 6(a). In our work, for convenience, we always use the diamond Brillouin zone for the honeycomb lattice. For the square lattice, the primitive lattice vectors are 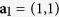 and 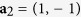. The primitive reciprocal lattice vectors are 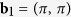 and 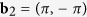. The square lattice has a square Brillouin zone as shown in Fig. 6(b). The brick-wall lattice can be considered a special honeycomb lattice with the bond angle 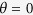. The primitive lattice vectors become 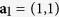 and 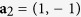. The primitive reciprocal lattice vectors are 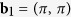 and 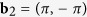. The corresponding Brillouin zone turns into a square, which is the same with that of the square lattice as shown in Fig. 6(b).

## Additional Information

**How to cite this article**: Hou, J.-M. and Chen, W. Hidden symmetry and protection of Dirac points on the honeycomb lattice. *Sci. Rep.* **5**, 17571; doi: 10.1038/srep17571 (2015).

## Supplementary Material

Supplementary Information
