# Supplementary material for: Hidden symmetry and protection of Dirac points on the honeycomb lattice
Source: Sci Rep. 2015 Dec 7;5:17571. doi: 10.1038/srep17571 (PMC4671008; doi:10.1038/srep17571)
Supplement: Supplementary Information [file srep17571-s1.pdf]

Supplementary information for
Hidden symmetry and protection of Dirac points on the honeycomb lattice

Jing-Min Hou[1] and Wei Chen[2]
[1]Department of Physics, Southeast University, Nanjing 211189, China
[2]College of Science, Nanjing University of Aeronautics and Astronautics, Nanjing 210016, China

S-1. FAILURE OF THE TIME-REVERSAL AND INVERSION SYMMETRIES PROTECTING THE DIRAC POINTS ON THE HONEYCOMB LATTICE

Here we prove in rather general terms that time-reversal and inversion symmetries are not sufficient to guarantee the existence of Dirac points.

Firstly, one can not obtain the existence of the Dirac points from the time-reversal and inversion symmetries. Without loss of generality, the Bloch Hamiltonian of the honeycomb lattice can always be written in the form as

$$\begin{aligned} H(\mathbf{k}) &= h_0(\mathbf{k})I_2 + h_x(\mathbf{k})\sigma_x + h_y(\mathbf{k})\sigma_y + h_z(\mathbf{k})\sigma_z \\ &= \begin{pmatrix} h_0(\mathbf{k}) + h_z(\mathbf{k}) & h_x(\mathbf{k}) - ih_y(\mathbf{k}) \\ h_x(\mathbf{k}) + ih_y(\mathbf{k}) & h_0(\mathbf{k}) - h_z(\mathbf{k}) \end{pmatrix}, \end{aligned} \tag{S1}$$

where $\sigma_{x,y,z}$ are the Pauli matrices and I_2 is the 2×2 unit matrix; $h_i(\mathbf{k}), (i = 0, x, y, z)$ are real functions of the wave vector $\mathbf{k}$. The dispersion relation is $E_\pm(\mathbf{k}) = h_0(\mathbf{k}) \pm \sqrt{h_x(\mathbf{k})^2 + h_y(\mathbf{k})^2 + h_z(\mathbf{k})^2}$. Under the action of time-reversal operator Θ and inversion operator I, the Bloch Hamiltonian for any wave vector $\mathbf{k}$ is transformed, respectively, as

$$\Theta : H(\mathbf{k}) = H^*(-\mathbf{k}), \tag{S2}$$

and

$$I : H(\mathbf{k}) = \sigma_x H(-\mathbf{k})\sigma_x. \tag{S3}$$

Then, under the combined action of time-reversal and inversion symmetries, the Bloch Hamiltonian is transformed as

$$\Theta I : H(\mathbf{k}) = \sigma_x H^*(\mathbf{k})\sigma_x = \begin{pmatrix} h_0(\mathbf{k}) - h_z(\mathbf{k}) & h_x(\mathbf{k}) - ih_y(\mathbf{k}) \\ h_x(\mathbf{k}) + ih_y(\mathbf{k}) & h_0(\mathbf{k}) + h_z(\mathbf{k}) \end{pmatrix}. \tag{S4}$$

Comparing Eq.(S4) with Eq.(S1), we only obtain $h_z(\mathbf{k}) = 0$. The corresponding dispersion relations becomes $E_\pm(\mathbf{k}) = h_0(\mathbf{k}) \pm \sqrt{h_x(\mathbf{k})^2 + h_y(\mathbf{k})^2}$. However, the time-reversal and inversion symmetries can not guarantee that $h_x(\mathbf{k})$ and $h_y(\mathbf{k})$ simultaneously vanish at some point of the Brillouin zone. Therefore, the time-reversal and inversion symmetries can not ensure the system being gapless.

S-2. THE DERIVATION OF THE BLOCH HAMILTONIAN

A. The honeycomb lattice

For the general honeycomb lattice with the bond angle θ, the tight-binding Hamiltonian can be written as,

$$H_\theta = -\sum_{i\in A}[t_1 a_i^\dagger b_{i+\hat{d}_1} + t_1 a_i^\dagger b_{i+\hat{d}_2} + t_2 a_i^\dagger b_{i+\hat{d}_3}] + H.c., \tag{S5}$$

where a_i is the annihilation operator that destructs a particle in the Wannier state located at the site i in sublattice A and b_j is the annihilation operator that destructs a particle in the Wannier state located at the site j in sublattice B; the subscript $i \equiv (i_x, i_y)$ is the coordinate for the lattice sites; $\hat{d}_1 = (\cos\theta, \sin\theta), \hat{d}_2 = (-\cos\theta, \sin\theta), \hat{d}_3 = (0, -1)$ represent the unit vectors between the two nearest lattice sites; t_1 and t_2 are the amplitudes of hopping as shown in figure 1(a) in the main text. We take the Fourier's transformation to the annihilation operators as

$$a_{\mathbf{k}} = \frac{1}{\sqrt{N}}\sum_i a_i e^{-i\mathbf{k}\cdot\mathbf{R}_i^A}, \tag{S6}$$

$$b_{\mathbf{k}} = \frac{1}{\sqrt{N}}\sum_j b_j e^{-i\mathbf{k}\cdot\mathbf{R}_j^B}, \tag{S7}$$

where $\mathbf{R}_i^A$ and $\mathbf{R}_j^B$ represent the positions of lattice sites in sublattice lattice A and B, respectively. Substituting Eqs.(S6) and (S7) into Eq.(S5), we obtain

$$H_\theta = -\sum_{\mathbf{k}}[2t_1\cos(\cos\theta k_x)e^{i\sin\theta k_y}a_{\mathbf{k}}^\dagger b_{\mathbf{k}} + t_2 e^{-ik_y}a_{\mathbf{k}}^\dagger b_{\mathbf{k}}] + H.c. \tag{S8}$$

We define the two-component annihilation operator as $\eta_{\mathbf{k}} \equiv [a_{\mathbf{k}}, b_{\mathbf{k}}]^T$ and the Hamiltonian can be written as $H_h = \sum_{\mathbf{k}}\eta_{\mathbf{k}}^\dagger\mathcal{H}_h(\mathbf{k})\eta_{\mathbf{k}}$. Here, $\mathcal{H}_h(\mathbf{k})$ is the Bloch Hamiltonian of the honeycomb lattice model for the wave vector $\mathbf{k}$ as

$$\begin{aligned}\mathcal{H}_\theta(\mathbf{k}) &= -[2t_1\cos(\cos\theta k_x)\cos(\sin\theta k_y) + t_2\cos k_y]\sigma_x \\ &\quad +[2t_1\cos(\cos\theta k_x)\sin(\sin\theta k_y) - t_2\sin k_y]\sigma_y,\end{aligned} \tag{S9}$$

where σ_x and σ_y are the Pauli matrices. This Bloch Hamiltonian is Eq.(1) in the main text.

B. The square lattice

For the square lattice with a hopping-accompanying π phase, the tight-binding Hamiltonian can be written as

$$H_s = -\sum_{i\in A}[t_x a_i^\dagger b_{i+\hat{x}} + t_x a_i^\dagger b_{i-\hat{x}} + t_y e^{-i\pi}a_i^\dagger b_{i+\hat{y}} + t_y a_i^\dagger b_{i-\hat{y}}] + H.c., \tag{S10}$$

where $\hat{x}$ and $\hat{y}$ represent the unit vectors in the x and y directions, respectively; t_x and t_y are the amplitudes of hopping along the x and y directions, respectively. Taking the Fourier's transformation, we obtain the Hamiltonian as

$$H_s = -\sum_{\mathbf{k}}[2t_x\cos k_x a_{\mathbf{k}}^\dagger b_{\mathbf{k}} - 2t_y i\sin k_y a_{\mathbf{k}}^\dagger b_{\mathbf{k}}] + H.c. \tag{S11}$$

We rewritten the Hamiltonian as $H_s = \sum_{\mathbf{k}}\eta_{\mathbf{k}}^\dagger\mathcal{H}_s(\mathbf{k})\eta_{\mathbf{k}}$ with $\eta_{\mathbf{k}} \equiv [a_{\mathbf{k}}, b_{\mathbf{k}}]^T$. Then, the Bloch Hamiltonian for the square lattice model is

$$\mathcal{H}_s(\mathbf{k}) = -2t_x\cos k_x\sigma_x - 2t_y\sin k_y\sigma_y. \tag{S12}$$
